# Progress on Antiangiogenic Therapy for Patients with Malignant Glioma

**DOI:** 10.1155/2010/689018

**Published:** 2010-04-06

**Authors:** Manmeet S. Ahluwalia, Candece L. Gladson

**Affiliations:** ^1^Brain Tumor Neuro-Oncology Center, Neurological Institute, Cleveland Clinic, Cleveland, Ohio 44195, USA; ^2^Department of Solid Tumor Oncology, Taussig Cancer Institute, Cleveland Clinic, Cleveland, Ohio 44195, USA; ^3^Department of Cancer Biology, Lerner Research Institute, Cleveland Clinic, Cleveland, Ohio 44195, USA

## Abstract

Glioblastoma (GBM) is the most common primary brain tumor occurring in America. Despite recent advances in therapeutics, the prognosis for patients with newly diagnosed GBM remains dismal. As these tumors characteristically show evidence of angiogenesis (neovascularization) there has been great interest in developing anti-angiogenic therapeutic strategies for the treatment of patients with this disease and some anti-angiogenic agents have now been used for the treatment of patients with malignant glioma tumors. Although the results of these clinical trials are promising in that they indicate an initial therapeutic response, the anti-angiogenic therapies tested to date have not changed the overall survival of patients with malignant glioma tumors. This is due, in large part, to the development of resistance to these therapies. Ongoing research into key features of the neovasculature in malignant glioma tumors, as well as the general angiogenesis process, is suggesting additional molecules that may be targeted and an improved response when both the neovasculature and the tumor cells are targeted. Prevention of the development of resistance may require the development of anti-angiogenic strategies that induce apoptosis or cell death of the neovasculature, as well as an improved understanding of the potential roles of circulating endothelial progenitor cells and vascular co-option by tumor cells, in the development of resistance.

## 1. Introduction

Malignant gliomas include WHO grade IV gliomas, also known as glioblastomas (GBM), and WHO grade III gliomas referred to as anaplastic gliomas (AG) (anaplastic astrocytoma, anaplastic oligodendroglioma, and anaplastic oligoastrocytoma). GBM is the most common primary brain tumor occurring in the United States of America; approximately 10,000 new cases are diagnosed each year [1, 2]. In this review, we will focus on the status of antiangiogenic therapy for GBM, as these tumors characteristically show evidence of angiogenesis (neovascularization) on histologic examination. Despite recent advances in therapeutics, the prognosis for patients with newly diagnosed GBM remains dismal; the median survival is 15 months when treated with the current standard of therapy, which is a combination of maximal surgical resection followed by concurrent chemoradiation and six months of adjuvant temozolomide [3]. Most patients with GBM develop tumor recurrence after the above therapy, and many centers are now treating these patients with bevacizumab (a monoclonal antibody to vascular endothelial growth factor (VEGF) that was recently approved by the FDA). Although clearly of benefit to some patients, the majority die within 6-9 months after initiation of anti-VEGF therapy [4–6]. Other antiangiogenic agents also have been examined in phase I or II clinical trials for patients with GBM, and promising results have emerged; however, a statistically significant increase in overall survival has not been reported to date. It is now becoming apparent that tumors also can act to enhance their vasculature through other mechanisms such as “co-option” of the existing vasculature. The contribution of these processes to tumor vascularization and their potential effects on anti-angiogenic therapies is an emerging field of great interest.

## 2. The Biology of Brain Tumor Vascularization

GBMs are among the most vascular tumors known and hence the tumor-associated vasculature is an attractive therapeutic target [7]. It is now well established that tumors can promote the formation of new vessels through the process of angiogenesis. It is thought that when a tumor reaches a certain size, the requirements for oxygen and nutrients lead to the growth of new blood vessels [8, 9]. The neovasculature that is formed in GBM never completely matures; however, this leads to an atypical vasculature that is constantly undergoing remodeling. There also is evidence to suggest that prior to the triggering of the process of formation of new vessels, tumor cells can obtain the necessary nutrients and oxygen by “co-opting” existing blood vessels [10]. This process appears to occur in very small tumors and appears to be dependent on the microenvironment in the specific organ and perhaps on the tumor type. In transplanted C6 rat gliomas in the rat brain co-option of existing blood vessels by tumor cells occurred initially when the tumors were several mm in diameter, and this was followed by vascular regression and ultimately by the development of a neovasculature [10]. Moreover, the process of vasculogenic mimicry [11], in which tumor cells function as blood vessel lining cells, may contribute to the blood supply in malignant tumors. Most of the research concerning tumor vasculature has focused on the mechanisms that promote the formation of new blood vessels through the process of angiogenesis and it is these mechanisms that have been targeted in the development of antiangiogenic therapies. Currently, relatively little is known concerning the mechanisms underlying co-option of blood vessels and vasculogenic mimicry, the effects of anti-angiogenic therapies on these processes, or the role of these processes in the activity of, or resistance to, the anti-angiogenic therapies that have been developed. 

### 2.1. Angiogenic Growth Factors: Their Receptors and Function

The signaling of VEGF, a proangiogenic growth factor, is important for GBM angiogenesis and involves paracrine interactions between the glioma cells, and the inflammatory cells that secrete VEGF, and the tumor-associated endothelial cells (EC) that express receptors for VEGF (VEGFR) [9]. The VEGF gene family is composed of five members: VEGF-A, VEGF-B, VEGF-C, VEGF-D, and the placental-derived growth factor (PlGF) [10–12]. Of these, family members VEGF-A, VEGF-B, and PlGF are involved in vascular angiogenesis, whereas VEGF-C and -D regulate lymphatic angiogenesis [9]. VEGF-A was originally discovered as a factor that induces vascular permeability, and has since been shown to be an important EC mitogen [13, 14]. It belongs to the platelet-derived growth factor (PDGF) superfamily and is located on chromosome 6 [15]. Once secreted by the tumor cells, stromal cells or ECs, VEGF can be tethered in the extracellular matrix (ECM) due to an association with proteoglycans or glycosaminoglycans [16, 17].

The main receptors for VEGF-A are VEGFR1 and VEGFR2 [15]. These receptors are upregulated on the ECs in GBM as compared to the ECs of normal brain [18]. VEGFR-2 is a major mediator of the mitogenic and angiogenic effects of VEGF through its activation of the phosphatidyl inositol 3-kinase (PI3K)/protein kinase B (AKT) and RAF-MAPK kinase-(MEK-) extracellular signal-regulated (ERK) MAP kinase pathways [15]. 

Basic fibroblast growth factor (bFGF) is another proangiogenic growth factor that is upregulated in GBM, in which it is expressed focally by tumor cells and also is expressed by the vasculature [19, 20]. The receptors for bFGF include FGFR1, FGFR2, and FGFR4. FGFR1 is upregulated in GBM and is expressed by both the tumor cells and the tumor ECs. FGFR4 is expressed only by the tumor cells, and FGFR2 is not expressed in the tumors but it is expressed in the normal brain [21, 22]. As is the case for VEGF, once secreted by the tumor cells, stromal cells or ECs, bFGF can be tethered in the ECM due to an association with proteoglycans or glycosaminoglycans [16, 17]. Binding of bFGF to its receptor results in activation of the protein kinase C*α* (PKC*α*) pathway [23, 24] and the ERK pathway [23, 25].

In addition to VEGF and bFGF, several other proangiogenic molecules have been implicated in the initiation or amplification of angiogenesis in GBM including stem cell factor (SCF) and interleukin-8 (IL-8), hepatocyte growth factor and urokinase [26–30]. SCF mediates its signals through activation of the c-KIT receptor and may be of particular interest in GBM as its overexpression is associated with a shorter survival in patients with malignant glioma [31]. IL-8 is a chemokine that is synthesized by macrophages, ECs (where it is stored in the Weibel-Palade bodies), and other cell types (such as epithelial cells) [32, 33]. IL-8 is released from macrophages as a result of a host inflammatory response and activates ECs through the chemokine-1 and 2 receptors (CXCR1 and CXCR2) [34].

Finally, in normal blood vessels, angiopoietin-1 (Ang-1) is expressed by pericytes, binds the Tie2 tyrosine kinase receptor expressed on the associated EC and signals for survival and stabilization of the blood vessel [35, 36]. In GBM there is increased expression of both Ang-1 and its antagonist angiopoietin-2 (Ang-2); Ang-2 is expressed by the ECs, whereas Ang-1 is expressed by the tumor cells and is not expressed by the tumor blood vessels [37]. This has led investigators to speculate that Ang-2 may have a proangiogenic function in the neovasculature of tumors. Of note, Ang-2 expression has been implicated in the co-option process as described below [10].

### 2.2. Proteolytic Degradation of the Basement Membrane, Endothelial Cell Sprouting, and Tube Formation

Proteolytic degradation of the EC basement membrane by matrix metalloproteinases (MMPs) exposes the ECs to ECM proteins that can regulate angiogenesis and promote EC movement or sprouting [11, 28, 31]. These cell-ECM interactions occur through specific receptors and binding partners expressed on the EC surface, such as integrin cell adhesion receptors that recognize specific ECM molecules [9, 28, 34, 38, 39]. In addition, integrin receptors cooperate, collaborate, or cross-talk with growth factor receptors in a specific manner to enhance the signaling of both the integrin and the growth factor receptor. For example, in the chick chorioallantoic membrane model of angiogenesis, integrins *α*v*β*5 and *α*v*β*3, which are expressed on the EC, bind ligands in the chorioallantoic membrane and promote EC survival, proliferation, and sprouting (migration) by cooperating with VEGFR2 and FGFR, respectively [39]. At the same time, activation of the platelet-derived growth factor (PDGF) receptor signaling pathway recruits pericytes to the new EC tube, where they deposit ECM proteins that aid in the formation of a new EC basement membrane, inducing the expression of fibronectin and nidogen-1 by the ECs [40–43]. Pericyte recruitment to the EC tube also promotes the upregulation of certain integrin receptors (*α*6*β*1, *α*3*β*1, *α*1*β*1, and *α*5*β*1) on the ECs that mediate the interaction of the EC with fibronectin, nidogen-1, and laminin in the new basement membrane [42, 43]. 

Pericytes are necessary for stabilization of the new EC tube. The finding that EC tubes lacking pericyte coverage become dilated [42] suggests that pericyte coverage of the EC tube is necessary for maintaining the appropriate vessel diameter. This concept is consistent with the dilated and tortuous blood vessels that are observed on histologic examination of many GBM tumors and suggests that this may be associated with the reduced pericyte coverage of blood vessels observed in these tumors [36, 44].

Endothelial progenitor cells (EPCs) from the bone marrow may also contribute to the neovasculature. EPCs are mobilized from the bone marrow by the cytokine stromal-derived factor-1*α* (SDF-1*α*) [45] that is expressed by angiogenic vessels in GBM, SDF1*α* binds to the G protein-coupled chemokine 4 receptor (CXCR4) expressed on circulating EPCs and also on tumor vessels [46]. A role for the SDF1*α*/CXCR4 signaling pathway in the development or maintenance of the GBM neovasculature is suggested by a report that demonstrated that the administration of a CXCR4 antagonist to an orthotopic xenograft mouse model of GBM inhibited tumor growth [47].

### 2.3. Abnormalities in the Neovasculature of GBM and Hypoxia

The failure of the GBM neovasculature to mature completely results in an atypical neovasculature that demonstrates excessive leakiness and lacks a normal blood brain barrier (BBB). Electron microscopic examination of the neovasculature in GBM has revealed that the tight junctions and adherens junctions (important contributors to the BBB) are abnormal and that the actin filaments associated with the junctions are disorganized [48]. These changes likely decrease the osmotic gradient between the vasculature and interstitium, elevating the interstitial fluid pressure in the tumor [49]. This has certain important clinical implications. First, the elevated interstitial fluid pressure can compromise drug delivery to the GBM. Second, the abnormalities in the neovasculature may enhance tumor cell access to the vasculature and aid in tumor cell migration and invasion along the EC basement membrane to previously uninvolved brain tissue. 

In addition, the neovasculature in GBM demonstrates prominent thrombosis, promoting local hypoxia within the tumor. This local hypoxia is exacerbated by the rapid growth of these tumors, which frequently results in an extensive necrotic core that further accentuates the hypoxic microenvironment. Hypoxia can promote tumor angiogenesis through activation of the transcription factor hypoxia-inducible factor -1*α* (HIF-1*α*) which, in turn, enhances production of proangiogenic growth factors. Normally, the von Hippel-Lindau (VHL) molecule inhibits the function of HIF-1*α* but hypoxia destabilizes VHL causing it to dissociate from HIF-1*α*. This results in HIF-1*α* binding to the hypoxia response element (HRE) in the promoter of several proangiogenic factors (i.e., VEGF and SDF-1*α*) thereby initiating their transcription [45, 50]. A second HIF family member, HIF-2*α*, is also activated by hypoxia and likely plays a role in promoting tumor angiogenesis in GBM [51]. Li and colleagues [51] have recently demonstrated that HIF-2*α* is preferentially expressed in glioma stem cells, in comparison to nonstem tumor cells and normal neural progenitors. Their work showed that HIF2*α*, not HIF1*α*, is selectively activated in glioma stem cells by hypoxia, inducing the expression of VEGF. In tumor specimens, HIF2*α* colocalized with markers of cancer stem cells. Furthermore, targeting HIF in glioma stem cells inhibited self-renewal, proliferation, and survival in vitro, and attenuated the tumor initiation potential of glioma stem cells in mouse xenografts. Therefore, HIF2*α* likely represents a promising new therapeutic target in patients with GBM [51].

### 2.4. Role of Cancer Stem Cells in Tumor Angiogenesis

In recent years, a minor population of cells has been identified in GBM and other malignant tumors that has characteristics of tumor-initiating cells. These cells have been referred to as cancer stem cells (CSCs) and in the case of GBM are referred to as glioma stem cells [52–54]. There is still some debate regarding the appropriate definition of CSCs, but an emerging consensus holds that these cells are capable of self-renewal, sustained proliferation, and initiating tumor formation when injected in very low numbers into an immunocompromised mouse host [55]. Of particular relevance to this review, a small but growing number of papers have suggested that CSCs promote angiogenesis in tumors. For example, Bao and colleagues (2006) demonstrated that conditioned media from glioma stem cells significantly promoted EC migration, proliferation, and tube formation as compared to conditioned media from nonstem glioma cells [56]. Consistent with this observation, Calabrese and colleagues (2007) showed that ECs interact closely with brain tumor stem cells in the perivascular location (this has been termed a vascular niche) and secrete factors that maintain these cells in a stem cell-like state [57]. Investigators have speculated that this vascular niche is an important target for therapeutic intervention, as disruption of the niche microenvironment can ablate the growth of CSCs and arrest tumor growth [57, 58]. It should therefore be borne in mind that this is a potential mechanism by which anti-angiogenic drugs could inhibit brain tumor growth [57]. Most recently, Folkins and colleagues (2009) compared the angiogenesis in tumor xenografts from C6 glioma cells containing either a low or a high fraction of CSCs, and found that CSC-high xenograft tumors demonstrated an increased microvessel density and blood perfusion, as well as inducing greater mobilization and recruitment of bone marrow-derived EPCs to the tumors [59]. Also, the CSC-high C6 cultures and xenograft tumors expressed higher levels of VEGF and SDF1, and blocking of these proangiogenic factors resulted in a reduction in the growth of the tumors compared to that observed with CSC low C6 cells [59]. These data suggest that CSCs do contribute to tumor angiogenesis by promoting both local EC activity and systemic angiogenic processes that involve the recruitment of bone marrow-derived EPC in a VEGF-and SDF1-dependent manner [59].

### 2.5. Role of Bone Marrow-Derived Progenitor Cells in Tumor Angiogenesis

There is evidence that the adult bone marrow plays a significant role in endothelial and lymphatic neovessel formation that supports tumor growth and invasion [60, 61]. As the brain lacks a lymphatic system, lymphatic neovessel formation is not relevant for GBM. The nonhematopoietic (CD45**^−^**) bone marrow-derived population of cells that likely contribute to tumor angiogenesis, bone marrow-derived EPCs, may directly contribute to the EC layer by merging with the wall of a growing blood vessel and differentiating into ECs, thereby providing an alternative source of ECs [62]. The circulating EPCs originally reported by Asahara et al. (1997) (defined as CD34^+/^ VEGFR2^+^) were shown to be capable of differentiating into an EC phenotype that expressed EC markers in vitro, and to be capable of incorporating into neovessels at sites of ischemia [62]. Shaked and colleagues (2006) showed that administration of a vascular disrupting agent, such as combretastatin, or chemotherapeutic drugs, such as Paclitaxel or 5-fluorouracil, to a mouse model of cancer resulted in the recruitment of bone marrow-derived EPCs to the tumor as well as severe tumor necrosis [63]. Other studies have suggested that circulating EPCs may contribute to tumor neovascularization in mouse models of cancer [64]. 

It should be noted that the contribution of circulating EPCs to neoangiogenesis has been questioned and the markers that identify bone marrow-derived EPCs have been debated vigorously [65, 66]. The reported level of incorporation of circulating EPCs into new blood vessels varies considerably and ranges in different studies from 5% to 50% [53, 63, 65, 67]. In addition, technical challenges exist in identifying vessel-incorporated bone marrow-derived EPCs. Most recently, Rafat and colleagues (2009) suggested that in 12 patients with GBM higher numbers of circulating EPCs appeared to correlate with a significantly higher tumor vessel density, as compared to patients with lower numbers of circulating EPCs [68]; however, this observation needs to be validated in a larger study. 

Bone marrow-derived hematopoietic cells have also been reported to contribute to tumor angiogenesis and invasion. Unlike the circulating EPCs discussed above, these cells are CD45^+^, and they include myeloid progenitor cells identified as GR1^+^CD11b^+^ in the mouse (in the human CD3^+^, CD14^+^, CD19^+^, CD57^+^ and HLA-DR negative) [69], CD11b^+^F4/80^+^ tumor-associated macrophages (TAMs) [70], Tie2-expressing monocytes [71], CXCR4^+^VEGFR1^+^ hemangiocytes [72], bone marrow-derived circulating cells that comprise a heterogeneous population of myeloid cells identified as CD45^+^CD11b^+^ in the mouse and human [73], platelet-derived growth factor receptor (PDGFR)^+^ pericyte progenitors [74], and vascular endothelial-(VE-) cadherin^+^ CD45^+^ leukocytes [75]. Supporting their potential importance in the response of the tumor to therapy, Shojaei and Ferrara reported that the recruitment of bone marrow-derived GR1^+^ myeloid cells resulted in a tumor that was refractory to treatment with inhibitors of VEGF [76]. In general, the above CD45^+^ bone marrow-derived cells are not thought to incorporate into the EC layer of the new vasculature; rather, some of the above cells are thought to incorporate into the tumor neovasculature as perivascular cells where they function to promote angiogenesis through paracrine mechanisms, such as local secretion of VEGF [73].

### 2.6. Co-Option of Existing Blood Vessels Occurs in Very Small Tumors

As discussed above, there is evidence to suggest that in small tumors, in which the neovasculature has not developed, the tumor cells obtain the necessary nutrients and oxygen needed for growth by co-opting existing blood vessels [10]. Of note, in transplanted C6 rat gliomas in the rat brain co-option of existing blood vessels by tumor cells occurred initially when the tumors were several mm in diameter, and this was followed by vascular regression and ultimately by the development of a neovasculature [10]. The same study also showed co-option of existing blood vessels with the propagation of rat mammary cancer cells in the rat brain and with the metastasis of Lewis lung cancer cells to the lung after intravenous injection. In each of these models, Ang-2 expression was upregulated at the co-opted blood vessels, suggesting a role for Ang-2 signaling in the co-option process [10]. Vessel co-option has been observed in several other tumors, including melanoma, ovarian carcinoma, and Kaposi sarcoma (reviewed in [11]). 

Another mechanism contributing to the blood supply in malignant tumors is vasculogenic mimicry [11]. In vasculogenic mimicry, tumor cells function as blood vessel lining cells and this has been described in several types of malignant tumors, including melanoma [11].

## 3. Clinical Use of Antiangiogenic Agents

### 3.1. VEGF Inhibitors

*Bevacizumab *is a monoclonal antibody directed toward VEGF that has become the prototype of anti-angiogenic agents in clinical use for treatment of GBM (see Table 1). In a phase II clinical trial, 68 patients with recurrent malignant glioma (33 AG and 35 GBM) were treated with bevacizumab and irinotecan in two cohorts (see Table 2). The combination therapy produced an impressive initial radiographic response rate of 57% for GBM and 61% for AG [5, 77]. This compared favorably with the benchmark response rate for temozolomide therapy at the first tumor recurrence, 5% for patients with recurrent GBM and 35% for patients with recurrent AG [78–80]. With the combination of bevacizumab and irinotecan the progression-free survival at six months (PFS-6) was 43% for recurrent GBM patients and 59% for AG patients, and this was an improvement over the accepted PFS-6 standard of 15% for GBM patients and 31% for AG patients treated with temozolomide [79, 80]. Therapy with bevacizumab and irinotecan also resulted in neurological improvement and a reduction or discontinuation in the use of corticosteroid treatment in 31% of patients. The regimen was well tolerated; only one CNS hemorrhage was reported in 68 patients treated, eight patients were taken off of the study for thrombotic complications (four patients with pulmonary embolism (PE), two with deep vein thrombosis, one with thrombotic thrombocytopenic purpura, and one with thrombotic stroke), and two patients died (one with PE and one with a thrombotic stroke). Other side effects reported included, proteinuria, fatigue, and gastro-intestinal toxicity; these have been described with bevacizumab therapy for other types of cancer. Other prospective and retrospective studies have demonstrated initial radiographic response rates of between 35% and 50% with the combination of bevacizumab and cytotoxic chemotherapy [4, 81–83]. 

As irinotecan has limited activity as a single agent, a phase II randomized clinical trial was performed to evaluate the benefit of the addition of irinotecan to bevacizumab. In this clinical trial 167 patients with recurrent GBM received either bevacizumab alone or bevacizumab in combination with irinotecan; there was no statistically significant difference in the median overall survival (OS) for bevacizumab therapy alone (9.2 months) when compared to the combination bevacizumab and irinotecan therapy (8.7 months) [6]. A recent study evaluated the approach of bevacizumab monotherapy in patients with recurrent GBM followed by irinotecan combined with bevacizumab [84]. In this study, 17/48 patients (35%) achieved an initial radiographic response and the PFS-6 was reported to be 29%. Addition of irinotecan to patients who progress on bevacizumab monotherapy failed to produce an objective radiographic response in any of the 19 patients [84]. Bevacizumab is presently being evaluated in the upfront setting with temozolomide and radiation in two randomized phase III trials sponsored by the Radiation Therapy Oncology Group and Hoffmann-La Roche [85, 86]. 

Other VEGF/VEGFR-targeted inhibitors include*, Aflibercept*, a soluble VEGF decoy or hybrid receptor that consists of portions of VEGFR-1 and -2 fused to an immunoglobulin G1 Fc region [87–89] and *Cediranib* (AZD2171), a pan-VEGFR tyrosine kinase inhibitor with activity against PDGFR and c-KIT [90] (see Table 1).

## 4. Multikinase Receptors Inhibitors (MTKI)

A number of MTKIs have been studied in GBM patients (see Tables 1 and 2) including*, Imatinib* [95–99], *Sorafenib *[100–102], *Sunitinib* [103], *Vandetanib* (ZD6474) [104–107], *Vatalanib* (PTK787) [91, 108–114], and *XL184* [92, 115–117].

## 5. Other Anti-Angiogenic Agents

Other anti-angiogenic agents evaluated in GBM (see Tables 1 and 2) include,* Thalidomide* [93, 118–123], *Lenalidomide* [124, 125], *Tamoxifen * [126–129], *Enzastaurin* (LY317615) [130, 131], and the integrin inhibitor *Cilengitide* [94, 132, 133].

## 6. Challenges in Evaluating the Response to Anti-Angiogenic Therapy with Imaging

The Macdonald Criteria have been used since 1990 to define response or progression in clinical trials of malignant glioma [134]. The Macdonald Criteria, based on WHO Criteria, utilizes measurement of the largest cross-sectional area of tumor on contrast-enhanced CT or MRI scan. Malignant gliomas can be irregular in shape, include large necrotic cavities, or be partially or completely nonenhancing, creating difficulty in accurately measuring the largest cross-sectional area on contrast-enhanced CT or MRI scan. In addition, the interpreter needs to take into account concurrent corticosteroid use and changes in neurological function. Thus, response evaluation based on finding a difference in the largest cross-sectional area of tumor on contrast-enhanced CT or MRI is even more difficult with anti-angiogenic therapy. 

Clinical trials with drugs that modify signal transduction through the VEGF signaling pathway (e.g., bevacizumab, and cediranib) can produce a rapid decrease in enhancement after initiation of therapy [5, 135], resulting in an apparent high response rate. Some of the changes observed on contrast-enhanced CT or MRI scan result from a rapid normalization of abnormally permeable blood vessels and are not due to an antitumor effect. Anti-angiogenic therapy likely decreases vascular permeability and restores at least in part the integrity of the BBB, this leads to less contrast leakage from the vasculature. There is evidence that VEGF pathway-targeted anti-angiogenic drugs likely alter the image characteristics of enhancing tumor more effectively than of nonenhancing tumor. Hence the extent of reduction in contrast enhancement may not reflect true antitumor activity of the anti-angiogenic agent. Not infrequently, the radiographic image observed after anti-angiogenic therapy suggests a radiographic response that is more impressive than the clinical benefit derived from the therapy. The term “pseudoresponse” has been used to define the situation wherein the contrast-enhanced MRI suggests an antitumor effect that does not correlate with a true clinical benefit [136]. Therefore, treatment response based on radiographic images is probably not an optimal end point for clinical trials evaluating anti-angiogenic agents. The international Response Assessment in Neuro-Oncology (RANO) Working Group has developed new criteria for evaluating tumor response in malignant gliomas that take into account both enhancing and nonenhancing tumor (best visualized on T2 and fluid-attenuated inversion recovery (FLAIR) MRI sequences) images [136]. This working group proposes that increased T2-FLAIR signal likely reflects growing tumor, such as when it appears outside of the radiation field, it produces mass effect, or it involves the cortical ribbon, and when it occurs in the absence of other potential explanation.

## 7. Resistance to Anti-Angiogenic Therapy

Although the anti-angiogenic therapy of patients with malignant glioma has resulted in a small increase in the PFS-6, in general these agents have failed to produce a sustained clinical response. For example, patients with malignant glioma treated with a VEGF inhibitor have shown temporary improvements, seen as reduced edema on imaging or tumor stabilization on imaging; however, the tumors ultimately progress after a brief response. In patients with tumor progression during treatment with bevacizumab, the downhill clinical course is often rapid, and may be fueled by discontinuing the agent. In a retrospective analysis, patients with malignant glioma who were treated with a second line regimen containing bevacizumab after failure of treatment with an initial therapy combination of bevacizumab and a cytotoxic agent had a median PFS of only 37.5 days [137]. Shaked and colleagues showed that treatment withdrawal of a vascular disrupting agent, such as combrestatin, to a mouse model of cancer resulted in the development of an aggressive and angiogenic tumor [63]. Supporting the concept that malignant tumors ultimately develop resistance to therapy with a VEGF inhibitor, two recent studies (one of which included a xenograft mouse model of GBM) suggested that treatment with a VEGF inhibitor alters the natural history of the tumor and promotes a highly invasive and metastatic phenotype [138, 139].

The anti-angiogenic therapy failures described above are thought to be due to the development of resistance to anti-angiogenic therapy. Resistance to anti-angiogenic therapy has been broadly classified as either adaptive or intrinsic [140]. Animal models of malignant glioma and of other malignant tumors have shown that tumors adapt to treatment with angiogenesis inhibitors by upregulating, or acquiring, an alternate mechanism(s) to sustain tumor growth, which has been termed “adaptive evasive resistance”. This can occur even when the specific target of the anti-angiogenic agent remains successfully inhibited. Adaptive resistance along with intrinsic resistance is thought to be the reason for the progression of malignant tumors treated with anti-angiogenic therapy. In tumors that show an initial response to anti-angiogenic therapy, adaptive evasive resistance is thought to be the main mechanism for the development of resistance, that is, blockade of one proangiogenic growth factor can lead to upregulation of an alternate proangiogenic growth factor [141]. For example, in a study of GBM patients treated with the pan-VEGFR inhibitor cediranib (AZD 2171), blood levels of the proangiogenic factors bFGF and SDF1*α* were noted to be higher in patients at the time of tumor progression or relapse as compared to the levels observed during the phase in which the patients showed a response to cediranib therapy [142].

Recruitment of vascular progenitor cells from the bone marrow may aid in the process of adaptive evasive resistance. Certain anti-angiogenic therapies can cause regression of tumor vessels resulting in hypoxia and lead to the recruitment of various bone marrow-derived progenitor cells. Bone marrow-derived progenitor cells (including both vascular progenitor cells and vascular modulatory cells) can be recruited through hypoxia-induced HIF1*α* activation resulting in the expression of the downstream effector SDF1*α* [45, 143–146]. GBM tumors with low HIF1*α* levels contain fewer bone marrow-derived cells and exhibit lower levels of angiogenesis and tumor growth, as compared to GBM tumors with high HIF1*α* levels [146]. This suggests that the recruitment of bone marrow-derived progenitor cells may constitute a mechanism for the development of adaptive evasive resistance to anti-angiogenic therapy. 

One reason for the initial response to bevacizumab therapy reported for some patients with GBM may be that the GBM neovasculature typically contains a reduced density of pericyte coverage [36]. Pericytes are important constituents of blood vessels in general and of tumor blood vessels, they provide prosurvival signals to ECs. ECs are thought to induce pericyte recruitment, thereby promoting their own survival. Two of the best characterized prosurvival signals for ECs are VEGF and the signals derived from pericyte association with ECs [36]. Tumor vessels lacking adequate pericyte coverage are more vulnerable to VEGF inhibition as they lack the pro-survival signal from pericytes [147]. Blood vessels covered with the normal density of pericytes probably survive therapy with a VEGF inhibitor as the pericytes can signal for EC survival or quiescence [148, 149].

Finally, the possibility of the development of an alternative mechanism, such as co-option, for tumor cells to acquire oxygen and nutrients must be considered in terms of resistance. Notably, in an orthotopic mouse model of GBM treated with a VEGFR selective kinase inhibitor or with a multitarget VEGFR kinase inhibitor, tumor progression (growth) ultimately occurred that was highly invasive. Perivascular tumor invasion similar to tumor co-option of blood vessels was observed at autopsy. Moreover, two other recent studies have suggested that tumor co-option of pre-existing blood vessels can support a more invasive tumor cell phenotype and tumor growth after VEGF- or VEGFR2-targeted therapy [150, 151]. As Ang-2 signaling has been implicated in mediating blood vessel co-option in the untreated early C6 rat glioma model of malignant glioma [10], human GBM tumors were immunostained for Ang-1 and Ang-2 and it was reported that Ang-2 expression was upregulated at tumor co-opted blood vessels. Whether the co-opted blood vessels that were observed post anti-angiogenic therapy also showed upregulation of Ang-2 remains to be determined.

The second mechanism of resistance to anti-angiogenic therapy that has been suggested is an intrinsic or inherent resistance [140]. This concept stems from the observation that a minority of patients do not appear to respond to anti-angiogenic therapy. It has been suggested that this may be due to the pre-existing activation of multiple proangiogenic signaling pathways or a pre-existing inflammatory cell infiltrate that provides a source of tumor VEGF resulting in vascular protection [140].

## 8. The Blood-Brain Barrier (BBB) and the Challenges of Drug Delivery

Drug concentrations within the central nervous system are dependent on multiple factors that include, the permeability of the agent across the BBB, the extent to which it is actively transported out of the brain, and the volume of distribution in the brain parenchyma [152]. The BBB is an anatomic- physiologic barrier that is formed by multiple components, including tight junctions between endothelial cells, pericytes, and the astrocytic foot processes. This barrier selectively allows entry of some substances, such as glucose, lipid-soluble molecules, and oxygen, while preventing entry of other substances. It is important to understand that although the BBB may be disrupted in some areas of a GBM, a substantial proportion of glioma cells can be located in areas with an intact BBB. The magnitude of tumor vascular permeability varies within these tumors, with the greatest permeability being found in the tumor core and a relatively intact BBB at the proliferating tumor edge. The presence of an intact BBB in some areas of the tumor and the presence of a partially functional BBB in other areas of the tumor can prevent the effective delivery of active therapeutic compounds. 

The BBB expresses high levels of drug efflux pumps such as P-glycoprotein (P-gp), breast cancer-resistance protein (BCRP), and other multiple drug resistance proteins (MRPs) that actively remove chemotherapeutic drugs from the brain [153]. Efflux transport systems such as by P-gp and the MRPs at the brain capillary ECs may play a role in limiting the passage of therapeutic agents across the BBB [154]. For example, the brain distribution of the tyrosine kinase inhibitor imatinib is reduced by active efflux via the P-glycoprotein [155]. The BCRP drug efflux transport pump is expressed in a number of normal tissues, in addition to the BBB [156]. Modulation of drug transporters represents a new potential strategy to improve efficacy of targeted agents. Anti-angiogenic agents such bevacizumab that mainly target the EC on the luminal side of the vessel may not depend on the ability to cross the BBB; however, this remains a concern for tyrosine kinase inhibitors like imatinib.

## 9. Biomarkers of Angiogenesis

Currently, there are no validated biomarkers to monitor the progress or response to anti-angiogenic therapy in patients with malignant glioma or other cancers [157]. Two promising biomarkers for assessment of angiogenesis in malignant tumors are the number of circulating endothelial cells (CECs) and the number of circulating endothelial progenitor cells (EPCs). A growing body of literature suggests that the number of CECs and circulating EPCs is significantly elevated in patients with different types of cancer [65]. Furthermore, in a small number of preclinical animal studies successful antitumor response to anti-angiogenic therapy was correlated with changes in the number of CECs and circulating EPCs [158]. Rafat and colleagues have evaluated the number of circulating EPCs in the blood of patients with malignant glioma, and reported that they were higher in patients with GBM as compared to healthy volunteers [68]. Higher tumor blood vessel densities were noted in the patients with GBM having higher numbers of circulating EPCs as compared to those patients with lower numbers of circulating EPCs [68]. This supports the further evaluation of circulating EPCs as a novel biomarker for the assessment of angiogenesis and for the assessment of the response to anti-angiogenic therapy in patients with GBM. 

Measurement of VEGF plasma levels may not be a generally useful biomarker of tumor angiogenesis. For example, baseline plasma VEGF levels are not correlated with survival outcome for patients with metastatic colorectal cancer or for patients with metastatic nonsmall cell lung cancer [159, 160]. As expected, the plasma level of VEGF in patients with GBM and brain metastases is elevated as compared to normal healthy volunteers. In patients with metastatic breast cancer higher plasma levels of VEGF were associated with shorter time of progression [161]. Thus, additional studies need to be performed to determine whether the measurement of plasma VEGF levels is useful in monitoring the response to anti-angiogenic therapy in general and to VEGF inhibitor therapy. As previously noted, blood levels of bFGF and SDF1*α* were noted to be higher in patients at the time of relapse as compared to levels observed in the response phase in patients with recurrent GBM treated with cediranib [142].

## 10. Unanswered Questions

Despite the remarkable progress in our understanding of the process of angiogenesis and the potential promise of targeting these processes for the treatment of GBM, several critical questions need to be addressed. These include questions regarding the fundamental processes involved in GBM vascularization and their role in the failure of therapy. Pressing questions in this category are: (1) are the GBM tumors in patients that have failed bevacizumab or other anti-angiogenic therapy avascular, or are the tumors co-opting the existing blood vessels to obtain the oxygen and nutrients needed?, (2) is the angiopoietin signaling pathway driving a blood vessel co-option process in human GBM that have failed therapy with a VEGF inhibitor or other anti-angiogenic agent?, and (3) do cancer stem cells (or glioma stem cells) promote angiogenesis in malignant gliomas and could we target them specifically with novel therapy?

There also is an urgent need to improve the ability to assess the effects of the anti-angiogenic therapies on angiogenesis (rather than tumor growth). Questions in this category include: (1) does the number of CECs and circulating EPCs in patients with tumors correlate with the tumor grade, and could their number be used to monitor anti-angiogenic therapy? (Phenotyping circulating EPCs with a comprehensive set of endothelial, progenitor, and hematopoietic markers should be performed to address the issue of what are the appropriate markers to be used to identify circulating EPCs in patients [65]), (2) what is the contribution of circulating EPCs to angiogenesis in untreated virgin tumors?, and (3) could a biomarker be identified that would aid in identifying adaptive evasive resistance (escape pathways) that should be targeted when a patient's tumor develops resistance to anti-angiogenic therapy?

## 11. Conclusions

Anti-angiogenic therapy appears to be a promising and novel approach for the treatment of malignant brain tumors. Clinical trials have shown improvement in the short-term progression-free survival. The response of patients with GBM to therapy with a VEGF inhibitor likely depends, at least in part, on whether the tumor neovasculature contains a normal density of pericytes, how capable the tumor is in co-opting pre-existing blood vessels, and whether previously co-opted blood vessels exist in the tumor. A better understanding of the mechanisms of resistance to anti-angiogenic therapy is needed such that we can improve our treatment strategies for these patients. The development and optimization of biomarkers to measure angiogenesis in tumors, such as quantitation of the numbers of CECs and circulating EPCs, will potentially help us identify patients that are responding to, or failing, anti-angiogenic therapy. Theoretically, these markers could also be used to identify the subset of patients that would benefit from anti-angiogenic therapy and thereby individualizing therapy for patients with malignant tumors, such as GBM. In addition, new anti-angiogenic therapies that induce apoptosis of the ECs in the neovasculature are needed, as this type of anti-angiogenic therapy may be less likely to induce therapeutic resistance.

## Figures and Tables

**Table 1:** Examples of antiangiogenic agents in clinical trial for patients with high grade glioma.

Drug	Type	Targets
ABT-510	Thrombospondin-1 mimetic peptide	CD36 receptor
AMG 102	Monoclonal antibody	HGF/SF
Aflibercept	Soluble decoy receptor	VEGF-A,B, PlGF
Bevacizumab	Monoclonal antibody	VEGF-A
Brivanib	Tyrosine kinase inhibitor	FGFR, VEGFR2
Cediranib	Tyrosine kinase inhibitor	VEGFR1–3, PDGFR*β*, c-Kit
Cilengitide	RGD synthetic peptide	Integrins *α*v*β*3, *α*v*β*5
CT-322	Fibronectin (adnectin)-based inhibitor	VEGFR1–3
Dasatinib	Tyrosine kinase inhibitor	PDGFR*β*, Src, BCR-ABL, c-Kit, EphA2
Imatinib	Tyrosine kinase inhibitor	PDGFR*β*, BCR-ABL, c-Kit
Lenalidomide	Immunomodulatory and anti-inflammatory	FGF pathway
Pazopanib (GW786034)	Tyrosine kinase inhibitor	VEGFR1–3, PDGFR*β*, c-Kit
Sorafenib	Tyrosine kinase inhibitor	VEGFR2,3, BRAF, PDGFR*β*, c-Kit, Ras, p38*α*
Sunitinib	Tyrosine kinase inhibitor	VEGFR2, PDGFR*β*, Flt3, c-Kit
Tandutinib (MLN518)	Tyrosine kinase inhibitor	PDGFR*β*, Flt3, c-Kit
Vandetanib (ZD6474)	Tyrosine kinase inhibitor	VEGFR2, EGFR, RET
Vatalanib (PTK787)	Tyrosine kinase inhibitor	VEGFR1–3, PDGFR*β*, c-Kit
XL-184	Tyrosine kinase inhibitor	VEGFR2, Met, RET, c-Kit, Flt3, Tie-2

A more complete listing of anti-angiogenic agents in clinical trials for patients with high grade gliomas can be found at the National Institutes of Health website http://www.clinicaltrials.gov/ when searching for “glioma, brain cancer, glioblastoma, and angiogenesis”.

**Table 2:** Selected clinical trials in patients with recurrent high grade glioma.

Agent	Phase	Diagnosis	Number of patients and Histology	Response Rate	PFS-6
Bev + Ir [5, 77]	II	Recurrent MG	68 (33 AG, 35 GBM)		43% GBM, 59% AG
Bev versus Bev + Ir [6]	II	Recurrent GBM	85 GBM (Bev) Vs 82 GBM (Bev +Ir)	RRR = 28% (Bev), RRR = 37% (Bev +Ir)	42% (Bev) versus 50% (Bev + Ir)
Aflibercept [89]	II	Recurrent MG	48 (16 AG, 32 GBM)	50% AG30% GBM	
Cediranib [90]	II	Recurrent GBM	16 GBM	56%	
Vatalanib [91]	I/II	Recurrent GBM	55 GBM	PR = 4%, SD = 56%	
XL184 [92]	II	Recurrent GBM	26 GBM	PR = 38%	
Thalidomide [93]	II	Recurrent MG	39 (14 AG, 25 GBM)	PR = 6%, MR = 6%, SD = 33%	
Cilengitide [94]	II	Recurrent GBM	81 GBM		16%

Abbreviations: Bev: bevacizumab, Ir: Irinotecan, PFS-6: progression free survival at 6 months, MG: malignant glioma, GBM: glioblastoma, AG: anaplastic glioma (includes anaplastic astrocytoma, anaplastic oligodendrogioma and anaplastic oligoastrocytoma), RR: response rate, RRR: radiological response rate, PR: partial response, SD: stable disease, MR: minor response, TMZ: temozolomide, and XRT: radiation.
